# Prediction of Plaque Progression in Coronary Arteries Based on a Novel Hemodynamic Index Calculated From Virtual Stenosis Method

**DOI:** 10.3389/fphys.2019.00400

**Published:** 2019-05-09

**Authors:** Kyung Eun Lee, Sung Woong Shin, Gook Tae Kim, Jin Ho Choi, Eun Bo Shim

**Affiliations:** ^1^Department of Mechanical and Biomedical Engineering, Kangwon National University, Chuncheon, South Korea; ^2^Bio-Convergence Technology Group, Korea Institute of Industrial Technology, Jeju, South Korea; ^3^SiliconSapiens Inc., Seoul, South Korea; ^4^Department of Cardiology, Samsung Medical Center, Sungkyunkwan University, Seoul, South Korea

**Keywords:** stenosis susceptibility index, virtual stenosis method, wall shear stress, fractional flow reserve, coronary plaque deposition

## Abstract

**Rationale:**

Predicting the sites in coronary arteries that are susceptible to plaque deposition is essential for the development of clinical treatment strategies and prevention. However, to date, no physiological biomarkers for this purpose have been developed. We hypothesized that the possibility of plaque deposition at a specific site in the coronary artery is associated with wall shear stress (WSS) and fractional flow reserve (FFR).

**Background and Objective:**

We proposed a new biomarker called the stenosis susceptibility index (SSI) using the FFR and WSS derived using virtual stenosis method. To validate the clinical efficacy of this index, we applied the method to actual pilot clinical cases. This index non-invasively quantifies the vasodilation effects of vascular endothelial cells relative to FFR variation at a specific coronary artery site.

**Methods and Results:**

Using virtual stenosis method, we computed maximum WSS and FFR according to the variation in stenotic severity at each potential stenotic site and then plotted the variations of maximum WSS (*y*-axis) and FFR (*x*-axis). The slope of the graph indicated a site-specific SSI value. Then we determined the most susceptible sites for plaque deposition by comparing SSI values between the potential sites. Applying this method to seven patients revealed 71.4% in per-patient basis analysis 77.8% accuracy in per-vessel basis analysis in percutaneous coronary intervention (PCI) site prediction.

**Conclusion:**

The SSI index can be used as a predictive biomarker to identify plaque deposition sites. Patients with relatively smaller SSI values also had a higher tendency for myocardial infarction. In conclusion, sites susceptible to plaque deposition can be identified using the SSI index.

## Introduction

Predicting what sites are susceptible to stenotic plaque deposition in coronary arteries is an important factor in the diagnosis of CAD. To date, several basic and clinical research studies have been conducted to examine the mechanism of plaque progression in coronary arteries. WSS, defined as the product of blood viscosity and the spatial gradient of blood velocity at the vascular wall, plays a critical role in plaque deposition within coronary arteries (Samady et al., 2011; Dolan et al., 2013; Nørgaard et al., 2016; Park et al., 2016; Ng et al., 2017; Hayashi et al., 2018). The contribution of WSS to the initiation and progression of atherosclerosis has been extensively studied by experimental and CFD methods (Samady et al., 2011; Nørgaard et al., 2016; Hayashi et al., 2018). These studies have revealed that coronary plaque deposition frequently happens near low WSS regions. Also, it has been shown that oscillatory WSS and a steep shear stress gradient can affect plaque progression. The molecular effects of WSS on plaque deposition at specific sites have also been widely investigated (Li et al., 2005; Lu and Kassab, 2011). However, despite the substantial progress in understanding the physiological and pathological mechanisms of plaque progression, only a few studies on what specific coronary artery sites are susceptible to coronary plaque generation or deposition have been published. Recently, Choi et al. (2015) proposed a hemodynamic index, called axial plaque stress, to assess the future risk for plaque rupture and to determine treatment strategies for patients with CAD. This index quantified the possibility of plaque rupture of coronary stenosis but was mainly limited to the evaluation of plaque rupture in an already narrowed coronary artery. In the absence of significant plaque deposition in coronary arteries, monitoring the future risk for coronary arterial stenosis is essential for early diagnosis. If it were possible to predict what sites in the coronary artery are more susceptible to plaque deposition, clinicians would be able to develop better treatment plans for their patients.

In this study, we predicted what specific coronary artery sites were vulnerable to plaque deposition in patients without severe coronary stenosis. We hypothesized that plaque deposition at a specific site of the coronary artery is associated with changes in WSS and FFR at the site according to stenosis severity. Then a novel biomarker, SSI, was developed based on these measures. To compute this index at the potential sites, we used the virtual stenosis method presented in our previous paper (Lee et al., 2016a). We validated the efficacy of the index by comparing the computed results with clinical observations in a pilot clinical study, and then assessed the utility of the index.

## Materials and Methods

**FIGURE 1:**
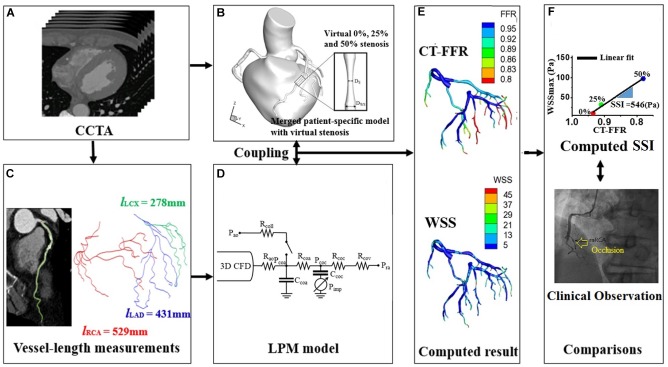
Schematic of computer simulation procedures used to obtain the SSI using the virtual stenosis model presented in our previous paper (Lee et al., 2016b). **(A)** The acquisition of coronary computed tomography angiogram, **(B)** merged process between patient-specific coronary model and virtual stenosis model, **(C)** measurements of vessel length from coronary computed tomography angiogram, **(D)** establishment of lumped parameter model, **(E)** computation of FFR and WSS, and **(F)** comparison between clinical observation and region with the minimum of computed SSI.

**FIGURE 2:**
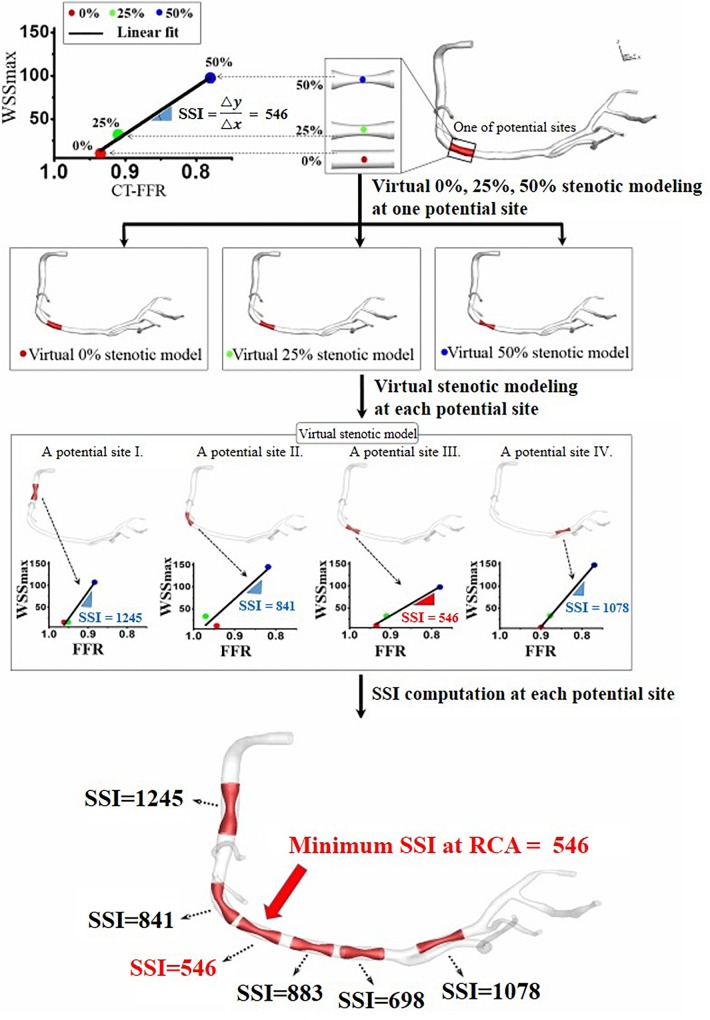
Site-specific SSI obtained from the linear relationship between computed WSS_max_ and FFR for three virtual stenotic models with stenotic severities of 0, 25, and 50% at a selected potential site.

The main procedures for SSI computation are shown in Figure 1. First, a three-dimensional (3D) patient-specific coronary model was reconstructed from CT images (Kwon et al., 2014; Lee et al., 2016a; Chung et al., 2017). We obtained CT image data from nine patients with suspicious CAD who underwent a medical examination at Samsung Seoul Medical Center in Seoul, Korea. The study was approved by the Institutional Review Board of the hospital. CT scans were performed in nine patients to observe stenotic severity in coronary arteries and the progression of stenosis. However, two patients were excluded due to severely calcified vessels that can result in incorrect image segmentation. CT image slices were 0.60 mm (512 × 512 pixels) in width and were segmented and reconstructed using a semi-automated method, as described in our previous paper (Kwon et al., 2014; Lee et al., 2016a,b; Chung et al., 2017). Each 0, 25, and 50% stenosed virtual model was merged into a potential site of the 3D patient-specific coronary model as Figure 2 (Lee et al., 2016a). To simulate coronary hemodynamics at hyperemia, we simulated unsteady blood flow behavior by using a Navier–Stokes equations solver in Eqs 1 and 2. Pulsatile blood flows in the 3D CFD coronary model at hyperemia were simulated by using Navier–Stokes solver which was based on segregated finite element method (Kwon et al., 2014) (Eqs 1 and 2).

(1) ∇⋅u=0

(2) ∂u∂t+u⋅∇u=−∇pρ+μρ∇2u

Here, ***u***, t, p, and ρ means velocity vector, time, pressure, and blood density, respectively.

To simulate coronary hemodynamics, we used a multi-scale simulation model by coupling patient-specific boundary conditions. The waveform of arterial blood pressure was used as an inlet boundary condition of the CFD model. Here, these pressures were based on the measured systolic/diastolic blood pressure and heart rate (Kelly and Fitchett, 1992; Verbeke et al., 2005; Laurent et al., 2006; Kwon et al., 2014). For the implementation of outlet boundary conditions of the CFD model, LPM is used to represent the physiological effects of micro-scale vessel effects. Collateral circulation was also considered in the lumped parameter model by using a switching system. On-off style switch works when coronary arterial distal pressure reaches a specific level (Schaper and Schaper, 1993; Seiler, 2009; Chung et al., 2017). Technical verification for the algorithm is shown in our previous paper (Kwon et al., 2014). For the identification of LPM resistance values, we used the vessel length based method that is explained in detail in our previous paper (Lee et al., 2016b, 2017). The method is based on the physiological observation that vessels with longer length feed more muscle mass inducing less microvascular resistance. In LPM models, the resistances of LAD and LCX are represented as follows (Lee et al., 2016b) (Eqs 3 and 4):

(3) RLAD=klLAD

(4) RLCX=klLCX

Here, *l* is the summed length exceeding a specific diameter and subscript represents the target vessel.

In the equations, resistance, denoted as “R”, are inversely proportional to vessel length, l, indicating that longer vessel length induces less resistance and thus more blood flow. Here, “k” is a proportional constant. In case of RCA, we divided the RCA vessels into two parts: the summed length of the RCA vessels feeding right ventricle, and the summed length of the RCA vessels feeding left ventricle. Since the summed length of the RCA vessels feeding right ventricle (RV) induces less blood flow than that of the RCA vessels feeding left ventricle (LV), the resistances of RCA are represented as follows (Lee et al., 2016b) (Eq. 5):

(5) RLCX=α k(lRCA)RV+k(lRCA)LV            (α> 1)

α is a constant (>1), reducing the effect of the RCA vessel length feeding RV muscle on RCA flow. Here, the value of α was set to be 3.45 which is a typical muscle volume ratio of LV to RV (Lee et al., 2016b). The proportional constant, k, in the equations, is derived from pressure-flow rate relation as explained in our previous paper (Lee et al., 2016b) (Eq. 6).

(6) $$k=\frac{\Delta P}{Q}\;\left\{l_{LAD}+l_{LCX}+\frac{{\left(l_{RCA}\right)}_{RV}\cdot {\left(l_{RCA}\right)}_{LV}}{{\left(l_{RCA}\right)}_{RV}+\alpha \cdot {\left(l_{RCA}\right)}_{LV}}\right\}$$

In the above equation, the Q and ΔP are the total flow rate to coronary arteries and the pressure difference between the aorta and coronary veins, respectively. A more detailed description of these methods can be found in our previous papers (Kwon et al., 2014; Lee et al., 2016a,b; Chung et al., 2017).

To compute an SSI value for coronary artery sites, we used the virtual stenosis method proposed in our previous paper (Lee et al., 2016a). In details, this method constructed the virtual stenosis models with 0, 25, and 50% of stenotic severity at a potential site, and each virtual stenosis configuration was merged into the 3D patient-specific coronary model as shown in Figure 2. We made the three virtual stenotic models for each coronary artery site to mimic plaque growth from mild to severe states. At each stenotic level at the site, maximum WSS (WSSmax) and FFR were computed and plotted to determine the correlation as shown in Figure 2. In this study, SSI was designated as the slope of the linear plot. Simply expressing, SSI is represented as follows (Eq. 7).

(7) $$\mathrm{SSI}=\;\frac{{\mathrm{WSSmax}}_{\mathrm{stenosis mode}}-{\mathrm{WSSmax}}_{\mathrm{non}-\mathrm{stenosis mode}}}{FFR_{\mathrm{non}-\mathrm{stenosis mode}}-FFR_{\mathrm{stenosis mode}}}$$

If we assume that WSSmax and FFR in non-stenosis model equal to zero, and one, respectively, then SSI can be expressed as (Eq. 8):

(8) $$\mathrm{SSI}=\;\frac{{\mathrm{WSSmax}}_{\mathrm{stenosis mode}}}{1-FFR_{\mathrm{stenosis mode}}}$$

After SSIs were computed for several potential coronary artery sites, we identified the location with the minimum SSI. We hypothesized that this location would be the most susceptible site for coronary artery stenosis. Therefore, we assumed this was where the patient was most likely to need PCI treatment due to a severe coronary artery occlusion. Based on this hypothesis, we computed SSI values from CT image data before the occurrence of severe coronary artery stenosis and identified the location with the minimum SSI value. Then we verified the validity of this hypothesis by comparing the location of the PCI with the location predicted using the smallest SSI.

## Results

**FIGURE 3:**
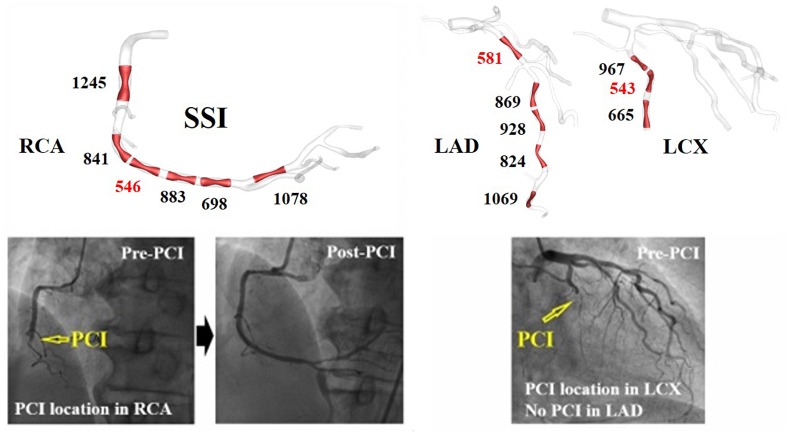
A vessel site-specific SSI and clinically observed stenosis progression in representative case I (Table 1).

The arterial site-specific SSI values from linear regression were obtained using three virtual stenotic models for a specific site, as shown in Figure 3. The minimum SSI values (546, 581, and 543) were observed in RCA, LAD, and LCX, respectively, as shown in Table 1. Of the minimum SSI values, the values obtained in the RCA and LAD were similar, and are marked with red arrows. MI was also found upon clinical observation in this patient. Furthermore, this patient was treated at the RCA and LAD sites marked with a yellow arrow. We found that the site where the minimum SSI was observed was co-localized with the PCI treatment site in the corresponding patient.

**Table 1:** Minimum SSI for each coronary artery, clinically observed MI, and matches between the minimum SSI and PCI site in nine vessels from seven patients.

	Minimum SSI per artery			Clinical observation	Match between the PCI and minimum SSI sites per artery		
			
	RCA	LAD	LCX	MI	RCA	LAD	LCX
Case I	**546**	581	**543**	Y	o	–	o
Case II	412	329	**239**	Y	–	o	o
Case III	792	874	**429**	N	–	–	o
Case IV	**729**	793		N	o	–	–
Case V	**468**	755	877	Y	–	×	–
Case VI	**520**	980	532	Y	–	×	–
Case VII	**1171**	1238	1400	N	o	–	–


*Bold values were minimum SSI value among three main coronary arteries (RCA, LAD, and LCX).*

Table 1 shows the minimum SSI value for each artery in all of the seven cases. The minimum SSI in the main arteries is written in bold. In five of seven patients, the site with the smallest SSI was identical to the site where PCI was performed due to stenosis progression, which equates to a 71.4% prediction rate. In seven of nine vessels, the site with the smallest SSI was also the site where PCI was performed, equating to a match rate of 77.8%. Good matches between PCI and minimum SSI sites are marked by a red circle. Mismatches are indicated using a black cross. The artery where PCI was not yet performed is marked using a hyphen (“-”). Furthermore, MI was observed in four of seven patients, as shown in Table 1. Compared to the absence of MI, the minimum SSI value of LAD tended to be smaller in cases with MI for all cases except VI.

## Discussion

Identifying sites that are susceptible to deposition is an important factor for determining the appropriate preventive therapy for CAD. Despite significant research in this area using both clinical and computational approaches, there are currently no biomarkers that allow for identification of specific sites at risk for plaque disposition. Therefore, we developed a new biomarker to predict the sites that are susceptible to coronary stenosis and evaluated the predictive capacity of this marker using pilot clinical data to confirm validity. The biomarker, in this case, is an index obtained using virtual stenosis method (Figure 1).

There are three main findings. First, we presented a new index called SSI. This index was able to predict which sites in the coronary arteries were more susceptible to plaque deposition. Applying this index to seven patients showed that we could estimate the actual PCI positions of the patients with 71.4 and 77.8% accuracy in per-patient basis and per-vessel basis, respectively (Table 1). Stenotic plaque in coronary arteries can easily accumulate in areas with low WSS such as recirculation zones, but to date, no index has been developed to quantify this. In this study, we proposed SSI to predict sites that were susceptible to plaque disposition and showed that this index could be used for the preventive treatment of patients.

Second, a comparison of SSI in the LADs of the seven patients showed that patients with low SSI in LAD were susceptible to MI (Figure 3). In clinics, stenosis of LAD causes MI, which induces severe damage to cardiac muscle. In this study, we obtained the minimum SSI values in the LADs of seven patients and investigated the relationship between the minimum SSI values and MI occurrence. Although the cut-off value of the minimum SSI value was not determined for all of the cases (only determined for case 6), the one reported MI occurred in the patient with the lowest SSI. Therefore, we can use the LAD SSI to predict the possibility of MI occurrence in a specific patient, resulting in a better-informed treatment plan for prevention. Thus, this index can become a useful clinical predictive index to prevent sudden death by MI.

Third, we presented a physiological hypothesis to show the efficacy of SSI. FFR decreases and WSSmax increases according to the increases of stenotic severity at specific sites. Here, the change in the rate of WSSmax relative to FFR change was defined as the SSI. Therefore, as shown in Figure 2, when the SSI was large at a specific site, WSS reached a critical value that could induce vascular vasodilation even after minimal increases in stenosis, thereby inhibiting plaque deposition. By contrast, if the SSI at a specific site was small, the critical WSS could be induced by more severe stenosis, and a stenotic plaque could easily accumulate at the location. A vasodilation effect can occur only when WSS is beyond a certain critical value; thus it is an important factor in reducing atherosclerosis, as evidenced by some physiological studies (Diamond et al., 1989; Ballermann et al., 1998; Chai et al., 2013).

However, there were still many limitations to this study. Most importantly, the pilot clinical data used in this study is small. Therefore, we have not been able to demonstrate the predictive ability of SSI in plaque progression or MI. Further validation will be the objective of future studies. Simulation of the data based on one or 2 years follow-up of patients with mild chest pain can provide the clinical evidence of SSI predictive ability in plaque progression. However, in the case of MI, because of the limited number of MI patients in one institution, SSI simulation study for large-scale retrospective data in multi-center basis will be a more plausible alternative.

Second, although the hypothesis in this study was physiologically reasonable, no *in vitro* or *in vivo* experiments were performed. Third, there are various factors (e.g., biochemical effects, vessel wall interaction, vasodilation effects, blood property effects, and lesion configurations effects) attributing to plaque progression. However, we hypothesized that the lumen geometrical factors might be a dominant factor in most cases. Furthermore, we used only symmetric plaque configurations without considering asymmetric plaque, various shapes of plaque, etc. These are also limitations in the current study. These diverse characteristics of plaque must be investigated in future research. Last, in this study, the occurrence of MI was predicted to increase when the SSI value was low, but data on the cut-off value were not available. Therefore, further in-depth studies using large-scale clinical data are required to provide criteria and data to determine the cut-off values. However, we believe that these limitations did not affect the major findings of the study.

## Ethics Statement

This study is non-invasive and uses only CT data of patients. This study was approved as a retrospective study by the Institutional Review Board of Seoul Samsung Medical Center. All methods were carried out in accordance with relevant guidelines and regulations. All subjects were over 18 years old, and informed consent was obtained from all subjects.

## Author Contributions

KL, JC, and ES provided the main idea of this research, analyzed the data, and wrote the manuscript. SS and GK assisted for technical supports. JC designed the clinical validation and provided the clinical images and data. All authors reviewed this manuscript. ES and JC contributed as corresponding authors.

## Conflict of Interest Statement

GK was employed by company SiliconSapiens Inc (Seoul, South Korea). The remaining authors declare that the research was conducted in the absence of any commercial or financial relationships that could be construed as a potential conflict of interest.

## Abbreviations

CAD: coronary artery disease
CFD: computational fluid dynamics
CT: computed tomography
FFR: fractional flow reserve
LAD: the left anterior descending artery
LCX: the left circumflex coronary artery
MI: myocardial infarction
PCI: percutaneous coronary intervention
RCA: the right coronary artery
SSI: stenosis susceptibility index
WSS: wall shear stress
